# Effect of water extract of bay laurel (*Laurus nobilis* L.) on non-alcoholic fatty liver disease (NAFLD)

**DOI:** 10.29219/fnr.v69.10668

**Published:** 2025-04-24

**Authors:** Minhee Lee, Jeongjin Park, Dakyung Kim, Seong-Hoo Park, Jaeeun Jung, Woojin Jun, Jinhak Kim, Kwang-Soo Baek, Ok-Kyung Kim, Jeongmin Lee

**Affiliations:** 1 Department of Food Innovation and Health, Kyung Hee University, Yongin 17104, Korea; 2 Division of Food and Nutrition and Human Ecology Research Institute, Chonnam National University, Gwangju 61186, Republic of Korea; 3 Department of Medical Nutrition, Kyung Hee University, Yongin 17104, Korea; 4 R&D Division, Daehan Chemtech Co. Ltd., Gwacheon 13840, Korea

**Keywords:** NAFLD, bay laurel leaves, lipid accumulation, inflammation, apoptosis

## Abstract

Non-alcoholic fatty liver disease (NAFLD) involves lipid accumulation in liver without consumption of alcohol and affects many people worldwide. NAFLD is associated with metabolic syndrome disease such as obesity, insulin resistance, hyperlipidemia, and diabetes. However, there are no pharmacologic therapies for NAFLD. Recently, there are increasing reports that several natural plants can inhibit lipid accumulation in hepatocytes. Bay laurel (*Laurus nobilis* L.) leaves have been used in traditional medicine for rheumatism, stomach ache, emetic, skin rashes, and earaches. Our objective was to investigate the effect of bay laurel leaves water extract (BLW) on free fatty acid (FFA) treated hepatocyte and high fructose, high fat (HFHF) diet in a mouse model of NAFLD. *In vitro*, lipid accumulation increased only in the FFA treated group, while BLW reduced lipid accumulation to a level comparable to that only in the FFA treated group. Cellular antioxidants were increased in the BLW compared to the only FFA-treated group, but cellular MDA levels were decreased in the BLW compared to the only FFA treated group. Cellular lipid accumulation, inflammation, and apoptosis were reduced in the BLW compared to the only FFA treated group. *In vivo*, serum ALT, AST, and GGT levels in the BLW supplementation group were significantly decreased compared with the HFHF group. Hepatic TC, TG, and MDA levels were significantly decreased in the HFHF+100 and HFHF+200 groups compared to the HFHF group. The hepatic antioxidant activities in the BLW supplementation groups were significantly increased compared to the HFHF group. The expression of proteins related to hepatic inflammation and apoptosis was reduced in the BLW supplementation groups compared to the HFHF group. These results suggest that BLW could be potentially useful in the treatment of NAFLD due to its inhibitory effects on hepatic lipogenesis, hepatic inflammation, and hepatic apoptosis.

## Popular scientific summary

- In vitro studies demonstrated that BLW significantly reduced lipid accumulation, oxidative stress, inflammation, and apoptosis in fatty acid-treated liver cells.
- In a mouse model of NAFLD induced by a high-fructose, high-fat (HFHF) diet, BLW supplementation improved liver function. This improvement was evidenced by lower levels of key markers of liver damage (ALT, AST, and GGT), reduced fat accumulation and oxidative stress, and suppressed inflammation and cell death in the liver.
- These results indicate that BLW may effectively inhibit liver fat accumulation, inflammation, and apoptosis, making it a potential therapeutic option for NAFLD.

Fatty liver is defined as the accumulation of more than 5% fat per weight in the liver. Fatty liver is caused by dyslipidemia, drugs, obesity, alcohol, Wilson’s disease, and hepatitis C. Non-alcoholic fatty liver disease (NAFLD) is defined as the accumulation of triglycerides (TG) in the liver without significant alcohol consumption. NAFLD is the most widespread liver disease in the world, and is associated with hepatocellular carcinoma, liver related events, and cardiovascular disease. Treatment of NAFLD is focused on lifestyle related factors such as body weight loss, increased physical activity, and dietary changes (1–4). Hepatic lipids are derived endogenously from dietary sources. Dietary lipids hydrolyze monoacylglycerols and free fatty acid (FFA) by pancreatic lipase. After lipid molecules are emulsified by bile acids, they are absorbed by enterocytes and resynthesized into TG. TG within chylomicrons are transported to the liver and FFA in plasma can be taken up by the liver and serves as an important substrate for TG synthesis in the liver. Several studies have reported the relationships between NAFLD and dietary factors such as a high-fat, high-fructose Western diet. FFA /fructose enter hepatocytes through transporters, particularly glucose transporter 2 (GLUT2) and cluster of differentiation 36 (CD36), which are highly expressed in the liver, accumulate there if excess levels of FFAs or fructose are present (5–7). Hepatic lipid accumulation causes mitochondrial dysfunction resulting in increased reactive oxygen species (ROS) production and oxidative stress. Excess accumulation of ROS activates nuclear factor-κB (NF-κB) pathways associated with the inflammatory factors, insulin receptor substrate 1 (IRS-1), and c-Jun N-terminal kinase (JNK) (8–12). Several studies have reported that natural foods can potentially protect against NAFLD. Bay laurel (*Laurus nobilis* L.) leaves are used a spice or flavoring agent in the culinary and food industries, and bay laurel leaves and fruits are used in traditional medicine for stomach complaints, rheumatism, and as a diaphoretic, and emetic agent. Bay laurel leaves contain flavonols, glycosylated flavonoid, alkaloids, and sesquiterpene lactones (13, 14). Our objective was to investigate the effects of bay laurel leaf water extract (BLW) on hepatic lipid accumulation, hepatic inflammation, and hepatic apoptosis pathways in high fructose, high fat (HFHF) diet induced NAFLD in mice as well as in hepatocyte using enzyme-linked immunosorbent assay (ELSIA) analysis, western blot analysis.

## Materials and methods

### Bay laurel (Laurus nobilis L.) leaves water extract preparation and quantitative analysis of isoquercetin

**Fig. 1:**
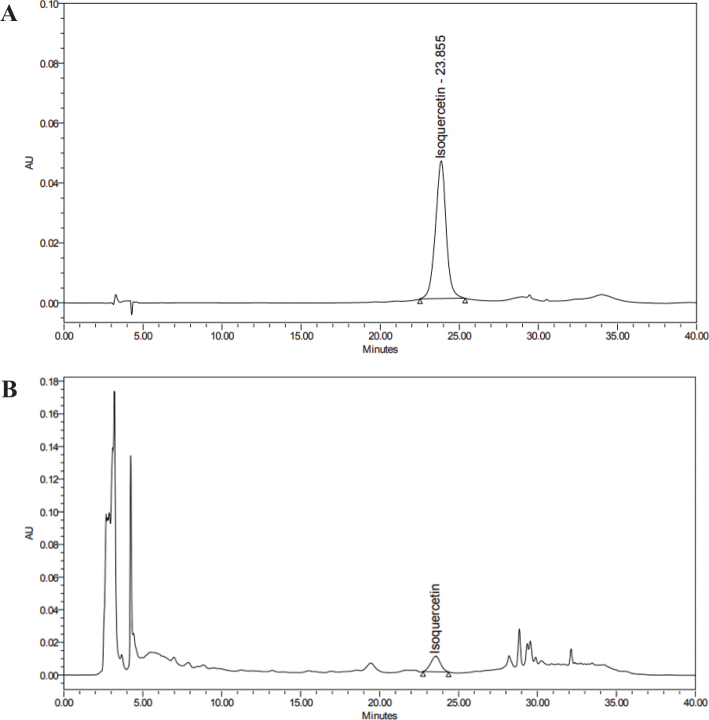
HPLC analysis of isoquercetin of BLW. (A) HPLC chromatogram of isoquercetin standard, (B) HPLC chromatogram of BLW.

Bay laurel (*Laurus nobilis* L.) leaves water extract powder (BLW) was obtained from Daehan Chemtech Co. Ltd (Gwacheon-si, Korea). The dried leaves were extracted twice with distilled water at 80°C and filtered. The extract was concentrated by vacuum drying. The levels of isoquercetin (IQ) in BLW were determined by high performance liquid chromatography (HPLC) (WATERS 2695 separations module, WATERS^©^, Milford, MA, USA). A column (Symmetry® C18, 5 μm, 4.6 mm × 250 mm) was used for the separation (1.0 mL/min, 30°C), the mobile phase was composed of water containing 0.3% phosphoric acid:acetonitrile = 85:15. BLW was standardized to contain at least 0.1% IQ (Fig. 1).

### Animals and treatment

The experimental protocols in this study were approved by the Institutional Animal Care and Use Review Committee of Kyung Hee University (KHUASP-20-348). C57BL/6J mice (male, 5 weeks old, 48 total mice) were obtained from Saeronbio, Inc. (Uiwang, Korea) and were housed in a standard environment (humidity of 50–60%, 22 ± 2°C, 12 h light/dark cycle). Mice were randomly divided into six groups; a normal AIN93G diet group (normal control;NC), a 10% high fructose+60% high fat diet group (HFHF), a 10% high fructose+60% high fat diet including 30 mg/kg body weight of silymarin extracts group (positive control; HFHF+PC), a 10% high fructose+60% high fat diet including 50 mg/kg body weight of BLW group (HFHF+50), a 10% high fructose+60% high fat diet including 100 mg/kg body weight of BLW group (HFHF+100), and a 10% high fructose+60% high fat diet including 200 mg/kg body weight of BLW group (HFHF+200). The concentrations of BLW in the diet were determined by the concentration used in the previous paper (15) and the concentration of silymarin was decided by converting the human supplementation concentration of silymarin approved by the Korea Food and Drug Administration. Mice were fed a high-fat diet supplemented with varying concentrations of Bay Laurel Water extract (BLW), based on the AIN93G formulation, and were additionally given 10% fructose solution via gavage. The concentrations of BLW, fructose, and fat in the diet were determined by the concentration used in the previous paper (14, 16). The mice were sacrificed after 12 weeks, and liver tissue and serum were collected for biochemical analyses and protein expression analysis.

### Histological observation (H&E staining)

Liver tissue of mice was removed, washed in phosphate buffer saline (PBS), fixed in 10% formalin, and decalcified. Blocks embedded in paraffin were sectioned into 7 μm thickness, stained with hematoxylin and eosin (H&E), and observed under an optical microscope.

### Cell culture and treatment

HepG2 cells were cultured in high-glucose Dulbecco’s modified Eagle’s medium (DMEM, HyClone Laboratories, Logan, UT, USA) supplemented with 10% fetal bovine serum (FBS, HyClone Laboratories) and 100 mg/L penicillin-streptomycin (HyClone Laboratories) at 37°C under a humidified atmosphere of 5% CO_2_ and 95% air. HepG2 cells were seeded at 5 × 10^5^ cells/well in 6 well culture plate and incubated for 24 h to induce excessive lipid accumulation in vitro in a hepatic steatosis model. HepG2 cells were pretreated with BLW solution (50, 100, and 200 μg/mL) and IG (10, 20 μm) for 4 h, and then 1 mM FFA mixture (2:1 ratio for oleate : palmitate) was added into each well. Subsequently, the cells were harvested for assay.

### Oil red O staining

HepG2 cells were pretreated with BLW solution (50, 100, and 200 μg/mL) and IG (10, 20 μM) for 4 h, and then 1 mM FFA mixture (2:1 ratio for oleate:palmitate) was added into each well. After 24 h, the cells were fixed with 10% formalin for 60 min, followed by staining with Oil Red O solution for 90 min. The cells were washed three times with PBS and then photographed. To evaluate the accumulation of lipid the cells were extracted using 100 % isopropanol, and the optical density was measured at 520 nm using an ELISA reader (Bio-Rad).

### ELISA assay

AST (Aspartate Aminotransferase Activity Colorimetric Assay Kit, Biovision), ALT (Alanine Aminotransferase Activity Colorimetric Assay Kit, Biovision), GGT (Gamma-glutamyl Transferase Colorimetric Assay Kit, Biovision), TC (Total Cholesterol Assay Kit, Biomax), TG (Triglyceride Quantification Kit, Biomax, Seoul, Korea), LDL/HDL-cholesterol (Biovision), SOD activity (Superoxide Dismutase Activity Kit, Biovision), CAT (Catalase Activity Kit, Biovision), GPx (Glutathione Peroxidase Activity Kit, Biovision), MDA (Lipid Peroxidation Colorimetric Assay Kit, Biovision) were measured in animal serum or liver, and HepG2 cells by ELISA. Experiments were performed according to the manufacturer’s instructions.

### Western blot analysis

Cells and liver tissues were lysed using the CelLytic^TM^ MT Cell Lysis Reagent (Sigma-Aldrich) to which the Halt^TM^ Protease & Phosphatase inhibitor Cocktail (Thermo Fisher Scientific, Rockford, IL, USA) had been added. Homogenized samples were centrifuged at 14,000 rpm at 4°C for 20 min. Protein content was quantified using the Bradford assay. Protein samples (50 μg each) were loaded onto 10% Mini-PROTEAN^®^ TGX^TM^ Precast Gels (Bio-Rad) and transferred to membranes using the Trans-Blot^®^ Turbo^TM^ Transfer system (Bio-Rad). Membranes were blocked with buffer (5% skim milk in Tris buffered saline with 1% Tween^®^ 20) for 1 h at room temperature. After washing, the membranes were exposed overnight at 4°C to the following primary antibodies : actin (1:3,000), GLUT2 (1:1,000), CD36 (1:1,000), Sirt1 (1:1,000), AMPK (1:1,000), phospho-AMPK (1:5,000), SREBP1c (1:1,000), ACL (1:1,000), phospho-ACL (1:500), ACC (1:1,000), phospho-ACC (1:500), FASN (1:800), PPAR-γ (1:100), CPT1A (1:1,000), IκB (1:800), phospho-IκB (1:500), NF-κB (1:800), phospho- NF-κB (1:500), COX-2 (1:1,000), iNOS (1:1,000). JNK (1:1,000), phosphor-JNK (1:500), Bax (1:1,000), Bcl-2 (1:1,000), Fas(CD95) (1:1,000), FADD (1:1,000), Cleaved-Caspase 8 (1:500), and Cleaved-Caspase 3(1:500). Washed membranes were incubated with secondary antibodies (anti-rabbits/mouse/Goat IgG HRP conjugated, 1:5,000) for 1 h at room temperature. Antibodies were purchased from Abcam (Cambridge, MA, USA), Bethyl (Montgomery, TX, USA), Cell Signaling Technology (Beverly, MA, USA), or LSbio (Seattle, WA, USA). Washed membranes were exposed to the luminol substrate EzWestLumi plus (ATTO, Tokyo, Japan) and luminescence was captured using Ez-Capture II equipment (ATTO). Images were analyzed using the CS Analyzer 3.0 software (ATTO).

### Statistical analysis

Statistical analysis was conducted using a specialized software (SPSS PASW Statistic v.25.0 software, SPSS Inc., Chicago, IL, USA) to evaluate the collected data. Numerical results were presented as averages with corresponding measures of dispersion. To assess differences between groups, a one-way analysis of variance was employed, followed by post-hoc testing to identify specific group distinctions. The threshold for statistical relevance was established at 5% (*P* < 0.05).

## Results

### Effects of BLW on the body weight and organ weights of a high fructose, high fat diet induced NAFLD in mice

The experimental animal’s body weight and organ weights changes were shown in Table 1. The animal weight was not significantly different among groups at the beginning of the experiment. HFHF mice showed a significant increase in both weight gain and FER compared to the NC group. The BLW supplementation groups showed a significant reduction in both weight gain and liver weight compared to the HFHF group. There were no significant differences in FER, kidney, and spleen weight among HFHF groups.

**Table 1:** Effects of BLW on body weight, weight gain, FER, and organ weights of high fructose, high fat diet induced NAFLD in mice

Groups	NC	HFHF	HFHF+PC	HFHF+50	HFHF+100	HFHF+200
Initial body weight (g)	22.34 ± 2.10^ns^	22.92 ± 0.95	23.58 ± 1.09	23.06 ± 0.64	23.65 ± 1.68	22.84 ± 1.64
Final body weight (g)	31.93 ± 2.56^c^	51.50 ± 2.84^a^	44.46 ± 4.01^b^	46.43 ± 5.87^b^	44.23 ± 2.67^b^	43.32 ± 4.22^b^
Weight gain* (g)	9.99 ± 1.3^c^	28.44 ± 2.67^a^	21.17 ± 4.50^b^	23.37 ± 5.67^b^	21.19 ± 3.11^b^	21.20 ± 4.95^b^
FER**	5.67 ± 1.30^b^	14.06 ± 1.92^a^	13.38 ± 2.73^a^	13.52 ± 3.28^a^	13.46 ± 2.37^a^	14.95 ± 3.47^a^
Organ weight (g)						
Liver	1.41 ± 0.17^b^	2.80 ± 0.81^a^	1.92 ± 0.44^b^	1.92 ± 0.51^b^	1.81 ± 0.57^b^	1.87 ± 0.53^b^
Kidney	0.33 ± 0.04^ns^	0.35 ± 0.04	0.36 ± 0.04	0.36 ± 0.04	0.34 ± 0.03	0.34 ± 0.03
Spleen	0.11 ± 0.03^ns^	0.12 ± 0.02	0.10 ± 0.03	0.12 ± 0.02	0.11 ± 0.02	0.11 ± 0.02

Values are presented as means ± SD values. Different letters indicate a significant difference at *P* < 0.05 as determined by Duncan’s multiple range test.

* Weight gain = final body weight (g) – initial body weight (g).

** FER = weight gain (g)/total food consumption (g) × 100.

**Table 2:** Effects of BLW on serum AST, ALT, GGT, lipid profiles, and hepatic TC and TG from high fructose, high fat diet induced NAFLD in mice

Groups	NC	HFHF	HFHF+PC	HFHF+50	HFHF+100	HFHF+200
**Serum**						
AST (mU/mL)	183.87 ± 30.81^c^	304.42 ± 32.62^a^	218.71 ± 56.43^bc^	254.19 ± 41.56^b^	197.51 ± 42.26^c^	187.53 ± 42.09^c^
ALT (mU/mL)	75.80 ± 3.22^b^	89.25 ± 6.72^a^	81.26 ± 3.11^b^	80.81 ± 6.29^b^	79.41 ± 5.15^b^	78.04 ± 6.96^b^
GGT (mU/mL)	90.98 ± 10.05^c^	161.16 ± 7.64^a^	107.50 ± 26.21^bc^	118.52 ± 25.90^b^	107.75 ± 4.91^bc^	95.16 ± 7.36^c^
Total cholesterol (μg/μL)	11.01 ± 6.52^b^	22.92 ± 4.94^a^	19.61 ± 3.74^a^	23.52 ± 2.71^a^	14.49 ± 1.91^b^	12.60 ± 1.74^b^
Triglyceride (μM)	12.08 ± 4.85^c^	41.04 ± 4.29^a^	29.16 ± 5.61^b^	37.96 ± 4.87^a^	31.91 ± 5.63^b^	29.45 ± 5.16^b^
HDL-chol (μg/μL)	46.99 ± 22.93^c^	116.79 ± 25.89^ab^	119.30 ± 22.47^ab^	107.51 ± 28.72^b^	133.23 ± 9.86^ab^	136.24 ± 30.87^a^
LDL/VLDL-chol (μg/μL)	28.85 ± 13.18^b^	91.83 ± 25.00^a^	37.11 ± 19.37^b^	32.65 ± 7.03^b^	26.39 ± 3.93^b^	25.84 ± 8.00^b^
HDL/LDL ratio	2.50 ± 0.75^d^	1.13 ± 0.37^e^	4.18 ± 0.92^bc^	3.36 ± 1.05^cd^	5.11 ± 0.60^ab^	5.44 ± 1.26^a^
**Hepatic**						
Total cholesterol (μg/mg tissue)	2.25 ± 0.16^c^	4.62 ± 1.11^a^	2.49 ± 0.36^c^	3.53 ± 0.26^b^	3.00 ± 0.32^bc^	2.39 ± 0.20^c^
Triglyceride (μM/mg tissue)	6.31 ± 1.33^c^	11.40 ± 1.40^a^	7.58 ± 0.58^bc^	10.19 ± 0.60^a^	8.72 ± 0.75^b^	7.58 ± 0.41^bc^

### Effects of BLW on liver tissue histology of high fructose, high fat diet induced NAFLD in mice

**Fig. 2:**
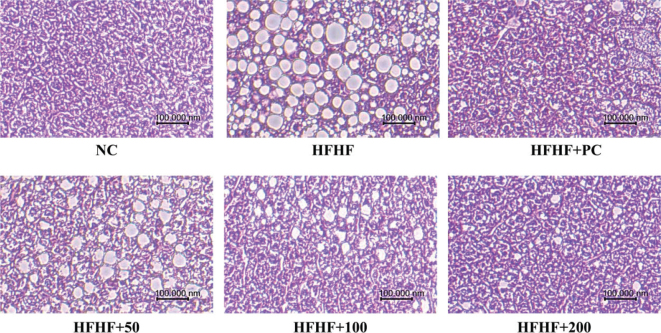
Effects of BLW on hepatic lipid accumulation using H&E staining of high fructose, high fat diet induced NAFLD in mice. NC, normal control; HFHF, 10% high fructose+60% high fat diet; HFHF+PC, 10% high fructose+60% high fat diet including 30 mg/kg body weight of silymarin extract; HFHF+50, 10% high fructose+60% high fat diet including 50 mg/kg body weight of BLW; HFHF+100, 10% high fructose+60% high fat diet including 100 mg/kg body weight of BLW; HFHF+200, 10% high fructose+60% high fat diet including 200 mg/kg body weight of BLW.

To investigate the inhibitory effect of BLW supplementation on hepatic lipid accumulation, we analyzed liver tissue using H&E staining. Histological analysis showed significant increases in hepatic lipid droplets in HFHF mice when compared with control mice (Fig. 2). BLW supplementation groups showed a significant reduction in hepatic lipid droplets compared with the HFHF group (Fig. 2).

### Effects of BLW on serum AST, ALT, GGT levels, and serum lipid profiles in high fructose, high fat diet induced NAFLD in mice

The experimental animal’s serum AST, ALT, GGT levels, and serum lipid profiles changes were shown in Table 1. Serum levels of AST, ALT, and GGT increased the most in the HFHF group, while BLW supplementation groups showed a significant reduction in serum ALT, AST, and GGT levels compared with the HFHF group. In particular, serum AST levels in the HFHF+100 group and HFHF+200 group were not significantly different from that in the NC group. Serum ALT levels in the BLW supplementation groups were not significantly different from that in the NC group. Moreover, serum GGT levels in the HFHF+100 and HFHF+200 groups were not significantly different from those in the NC group. Serum TC, TG, HDL-cholesterol, and LDL-cholesterol levels showed significant increases in the HFHF group compared to the NC group. Serum TC, TG, and LDL/VLDL-cholesterol levels of HFHF+100 and HFHF+200 groups were significantly lower than those of the HFHF group. Serum HDL-cholesterol levels were not significantly different among HFHF mice. However, the ratio of HDL-cholesterol to LDL-cholesterol was significantly increased in the BLW supplemented mice, compared with the HFHF group.

### Effects of BLW on hepatic TC and TG levels of high fructose, high fat diet induced NAFLD in mice

The experimental animal’s hepatic TC and TG changes were shown in Table 1. Hepatic TC and TG level was significantly increased in the HFHF group compared to the NC group, while BLW supplementation groups showed significant decreases in hepatic TC and TG levels compared with the HFHF group.

### Effects of BLW on hepatic antioxidant enzyme activities, MDA levels of high fructose, high fat diet induced NAFLD in mice

**Fig. 3:**
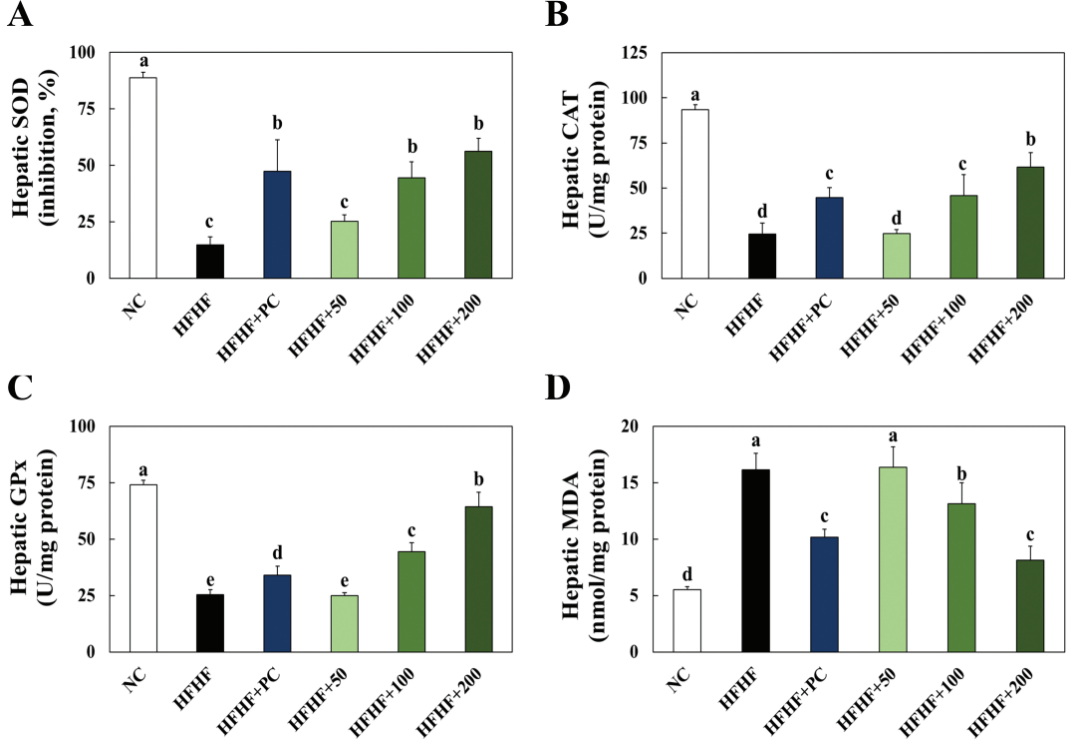
Effect of BLW on the hepatic SOD (A), catalase (B), GPx activities (C) and MDA levels (D) of high fructose, high fat diet induced NAFLD in mice. NC, normal control; HFHF, 10% high fructose+60% high fat diet; HFHF+PC, 10% high fructose+60% high fat diet including 30 mg/kg body weight of silymarin extract; HFHF+50, 10% high fructose+60% high fat diet including 50 mg/kg body weight of BLW; HFHF+100, 10% high fructose+60% high fat diet including 100 mg/kg body weight of BLW; HFHF+200, 10% high fructose+60% high fat diet including 200 mg/kg body weight of BLW. Values are presented as the mean ± standard deviation (*n* = 8), and different superscripted letters indicate significance at *P* < 0.05.

The experimental animal’s hepatic SOD, CAT, and GPx activity changes were shown in Fig. 3. Hepatic SOD, CAT, and GPx activity was significantly lower in the HFHF group compared to the NC group. However, BLW supplementation groups showed an increase in hepatic SOD, CAT, and GPx activity compared with the HFHF group (Fig. 3A–C). Hepatic MDA level was significantly higher in the HFHF group than the NC group, but BLW supplementation groups showed significant decreases in hepatic MDA levels compared with the HFHF group (Fig. 3D).

### Effects of BLW on hepatic lipid accumulation related protein expression in the liver of high fructose, high fat diet induced NAFLD in mice

**Fig. 4:**
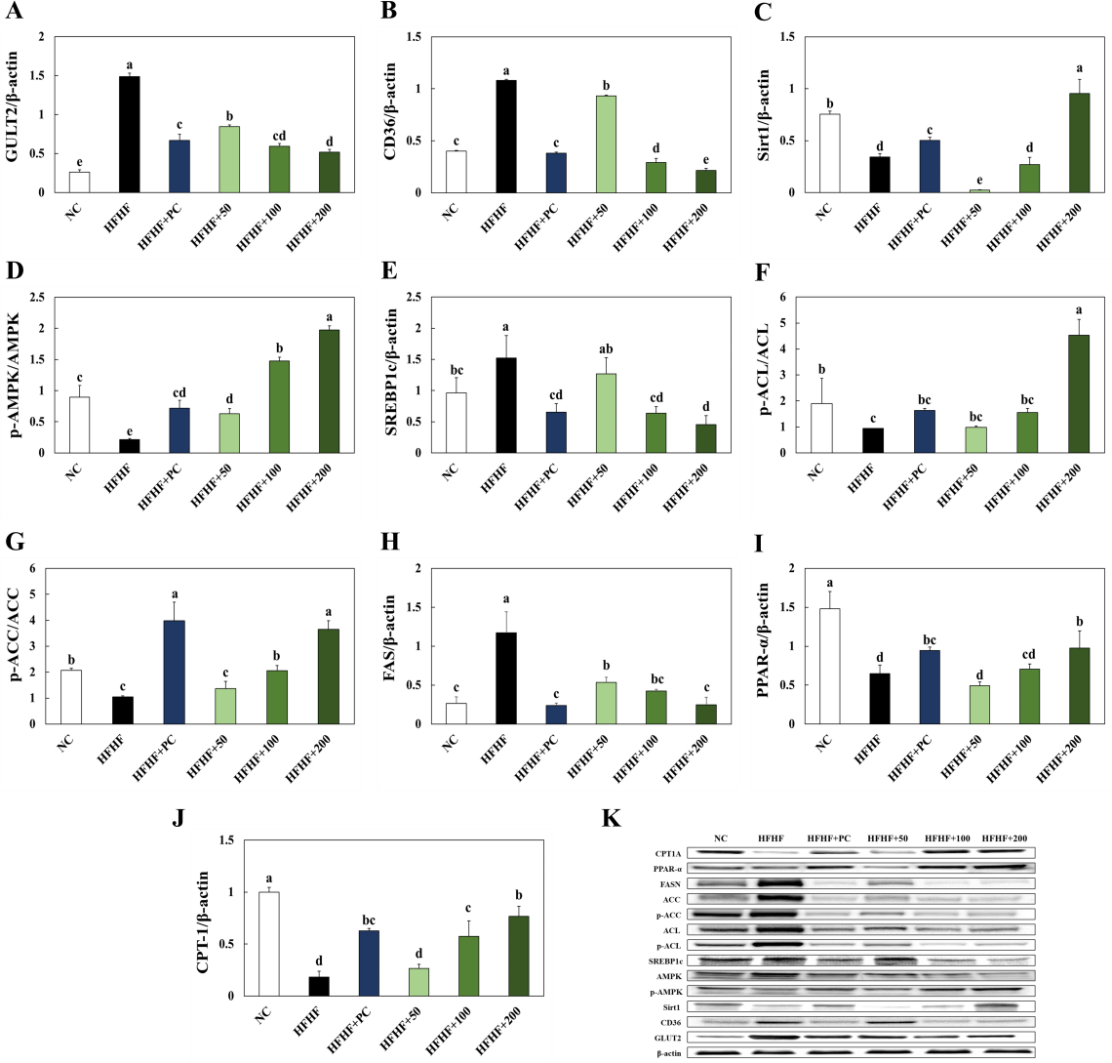
Effect of BLW on the protein expression of GLUT2 (A), CD36 (B), Sirt1 (C), p-AMPK/AMPK (D), SREBP1c (E), p-ACL/ACL (F), p-ACC/ACC (G), FAS (H), PPAR-α (I), CPT1A (J), and protein band (K) in liver of high fructose, high fat diet induced NAFLD in mice. NC, normal control; HFHF, 10% high fructose+60% high fat diet; HFHF+PC, 10% high fructose+60% high fat diet including 30 mg/kg body weight of silymarin extract; HFHF+50, 10% high fructose+60% high fat diet including 50 mg/kg body weight of BLW; HFHF+100, 10% high fructose+60% high fat diet including 100 mg/kg body weight of BLW; HFHF+200, 10% high fructose+60% high fat diet including 200 mg/kg body weight of BLW. Values are presented as the mean ± standard deviation (*n* = 8), and different superscripted letters indicate significance at *P* < 0.05.

The experimental animal’s hepatic lipid accumulation related protein expressions were shown in Fig. 4. To investigate the improvement effect of BLW supplementation on protein expression related to the hepatic lipid accumulation pathway, we analyzed lipid accumulation related protein expression levels in the liver tissue by western blot evaluation. GLUT2 and CD36 expressions were significantly increased in the HFHF group compared with the NC group. BLW supplementation groups showed a dose-dependent decrease in GLUT2 and CD36 expressions compared to the HFHF group (Fig. 4A, B). Sirt1 and p-AMPK/AMPK ratio expressions were significantly decreased in the HFHF group compared with the NC group, and the BLW supplementation groups showed a decrease in Sirt1 and p-AMPK/AMPK ratio expressions (Fig. 4C, D). SREBP-1c expression was significantly increased in the HFHF group compared with the NC group, while BLW supplementation groups showed a decrease in SREBP-1c expression (Fig. 4E). The p-ACL/ACL ratio and p-ACC/ACC ratio were significantly decreased in the HFHF group compared with the NC group, but BLW supplementation groups had a higher p-ACL/ACL ratio and p-ACC/ACC ratio than the HFHF group (Fig. 4F, G). FAS expression was significantly increased in the HFHF group compared with the NC group, but BLW supplementation groups showed a dose-dependent increase in FAS expression compared with the HFHF group (Fig. 4H). PPAR-α and CPT1A expressions were significantly lower in the HFHF group than the NC group, while BLW supplementation groups showed an increase in PPAR-α expression relative to the HFHF group (Fig. 4I, J).

### Effects of BLW on inflammation and apoptosis related protein expression in liver of high fructose, high fat diet induced NAFLD in mice

**Fig. 5:**
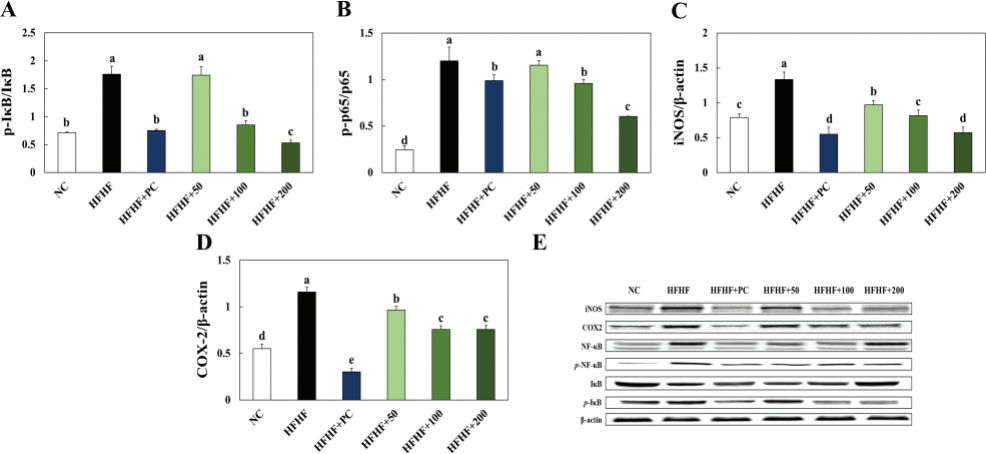
Effect of BLW on the protein expression of p-IκB/IκB (A), p-p65/p65 (B), COX-2 (C), iNOS (D), and protein band (E) in liver of high fructose, high fat diet induced NALFD in mice. NC, normal control; HFHF, 10% high fructose+60% high fat diet; HFHF+PC, 10% high fructose+60% high fat diet including 30 mg/kg body weight of silymarin extract; HFHF+50, 10% high fructose+60% high fat diet including 50 mg/kg body weight of BLW; HFHF+100, 10% high fructose+60% high fat diet including 100 mg/kg body weight of BLW; HFHF+200, 10% high fructose+60% high fat diet including 200 mg/kg body weight of BLW. Values are presented as the mean ± standard deviation (*n* = 8), and different superscripted letters indicate significance at *P* < 0.05.

**Fig. 6:**
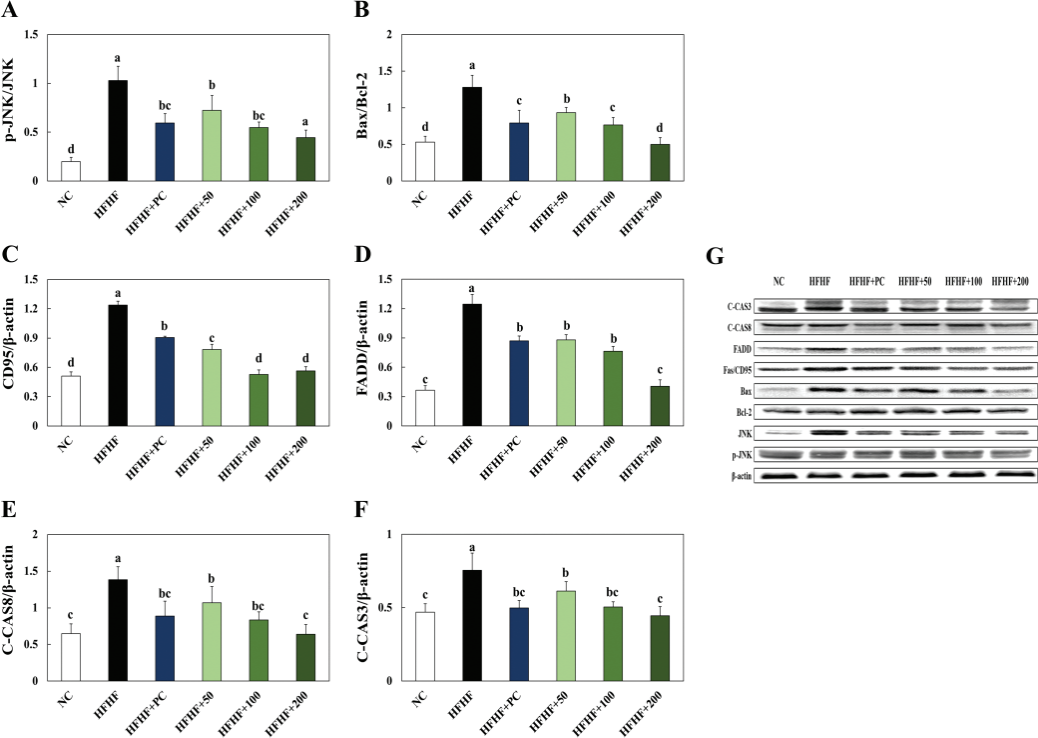
Effect of BLW on the protein expression of p-JNK/JNK (A), BAX/Bcl-2 (B), CD95 (C), FADD (D), C-CAS8 (E), C-CAS3 (F), and protein band (G) in liver of high fructose, high fat diet induced NALFD in mice. NC, normal control; HFHF, 10% high fructose+60% high fat diet; HFHF+PC, 10% high fructose+60% high fat diet including 30 mg/kg body weight of silymarin extract; HFHF+50, 10% high fructose+60% high fat diet including 50 mg/kg body weight of BLW; HFHF+100, 10% high fructose+60% high fat diet including 100 mg/kg body weight of BLW; HFHF+200, 10% high fructose+60% high fat diet including 200 mg/kg body weight of BLW. Values are presented as the mean ± standard deviation (*n* = 8), and different superscripted letters indicate significance at *P* < 0.05.

The experimental animal’s hepatic inflammation and apoptosis related protein expressions in were shown in Figs. 4, 5. The p-IκB/IκB ratio, p-p65/p65 ratio, COX-2, and iNOS expressions were significantly higher in the HFHF group than the NC group, while BLW supplementation groups showed a decrease in those expressions (Fig. 5). The apoptosis related proteins such as p-JNK/JNK ratio, BAX/Bcl-2 ratio, CD95, FADD, C-CAS8, and C-CAS3 were significantly increased in the HFHF group compared with the NC group. However, BLW supplementation groups showed a decrease in those protein expressions compared with the HFHF group (Fig. 6).

### Effect on BWL and IQ on lipid accumulation in FFA treated HepG2 cells

**Fig. 7:**
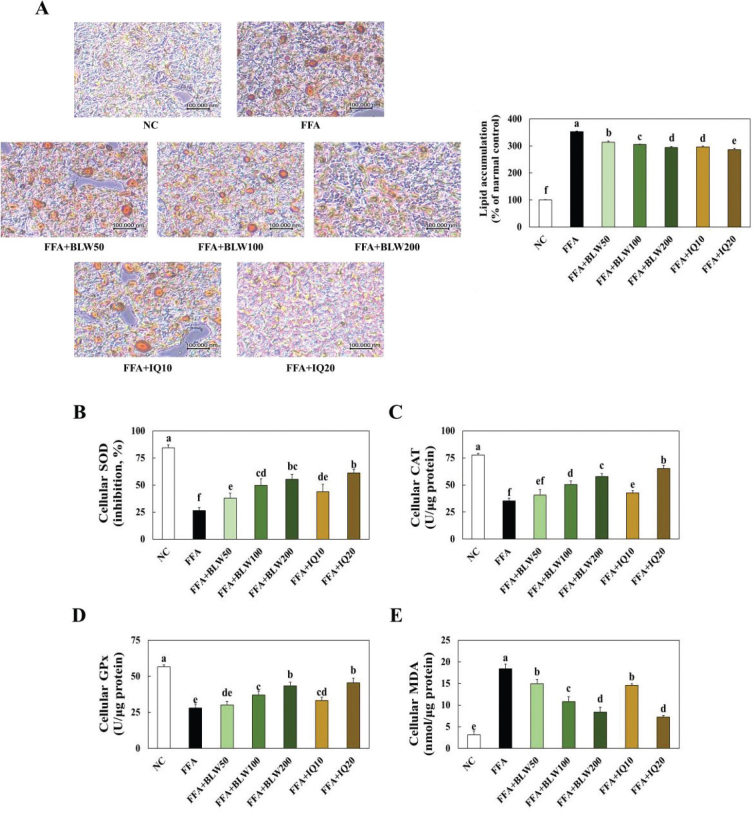
Effect of BLW and IQ on histological analysis using oil red staining and lipid accumulation (A), SOD (B), CAT (C), GPx (D), and MDA levels (E) in FFA treated HepG2 cells. NC, Normal control; C, 1 mM free fatty acid (FFA, 2:1 ratio for oleate:palmitate); FFA+BLW50, 50 μg/mL of BLW+1 mM FFA; FFA+BLW100, 100 μg/mL of BLW+1 mM FFA; FFA+BLW200, 200 μg/mL of BLW+1 mM FFA; FFA+IQ10, 10 μM of isoquercetin+1 mM FFA; FFA+IQ20, 20 μM of isoquercetin+1 mM FFA. Values are presented as the mean ± standard deviation (*n* = 3), and different superscripted letters indicate significance at *P* < 0.05.

To investigate the inhibitory effect of BLW and IQ on lipid accumulation, we conducted analysis of the HepG2 cells treated FFA using oil-red O staining (Fig. 7A). The oil-red O analysis showed significant increases in hepatic lipid droplets in the only FFA treated group compared to the NC group. The FFA treated with BLW treatment showed a significant reduction in lipid accumulation compared to the only FFA treated group. Also, FFA treated with IQ treatment showed a significant reduction in lipid accumulation compared to the control group. The SOD, CAT, GPx activities, and MDA levels were shown in Fig. 7B–E. The SOD, CAT, and GPx activities showed a significant decrease in the only FFA treated group when compared with NC group, while FFA treated with BLW and IQ treatment groups showed a significant increase compared to the only FFA treated group.

### Effects of BLW and IQ on protein expression of the de novo lipogenesis pathway in FFA treated HepG2 cells

**Fig. 8:**
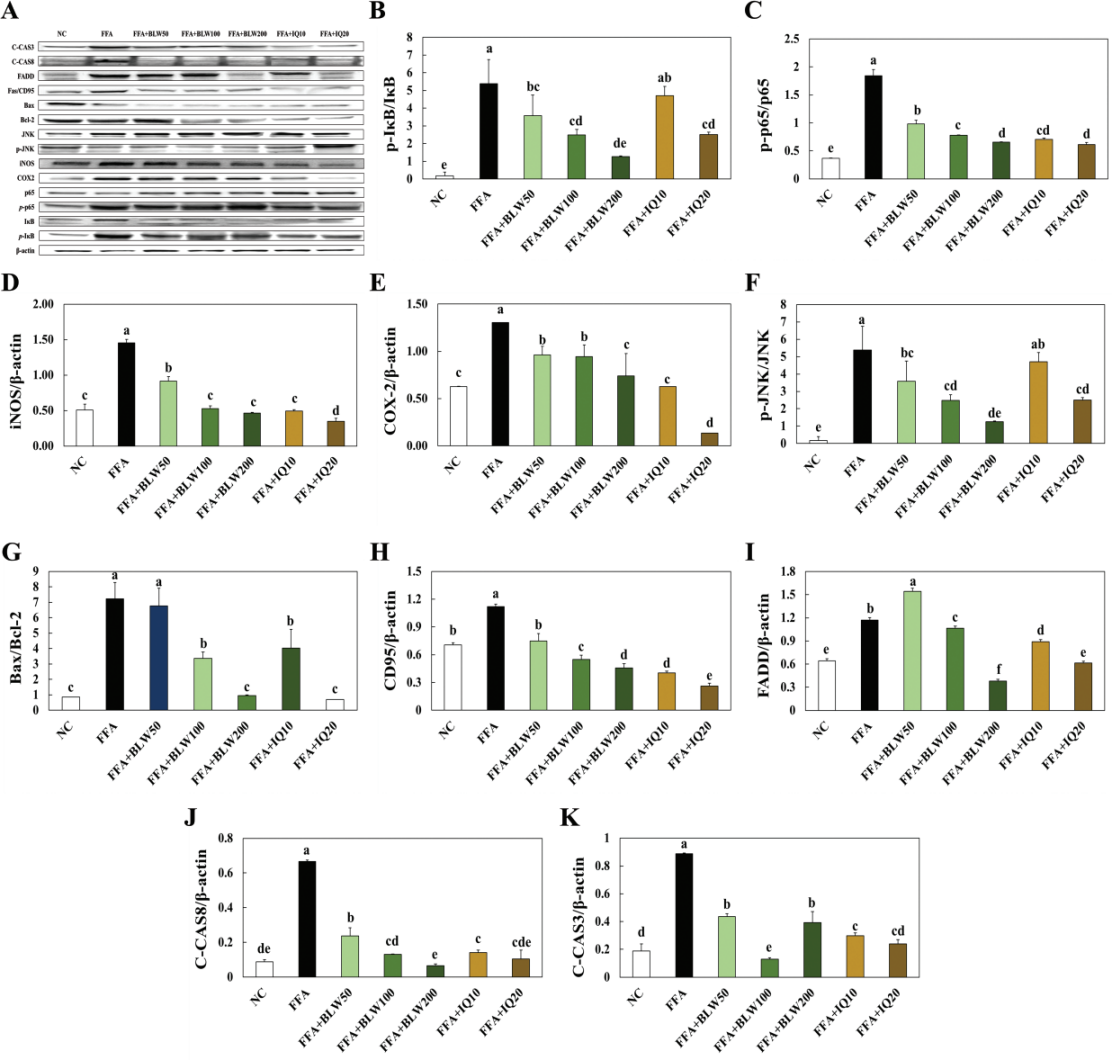
Effect of BLW and IQ on protein expression of de novo lipogenesis pathways in FFA treated HepG2 cell. (A) band, (B) CD36, (C) Sirt1, (D) p-AMPK/AMPK, (E) SREBP-1c, (F) p-ACL/ACL, (G) p-ACC/ACC, (H) FASN, (I) PPAR-α, (J) CPT1A. NC, Normal control; C, 1 mM free fatty acid (FFA, 2:1 ratio for oleate:palmitate); FFA+BLW50, 50 μg/mL of BLW+1 mM FFA; FFA+BLW100, 100 μg/mL of BLW+1 mM FFA; FFA+BLW200, 200 μg/mL of BLW+1 mM FFA; FFA+IQ10, 10 μM of isoquercetin+1 mM FFA; FFA+IQ20, 20 μM of isoquercetin+1 mM FFA. Values are presented as the mean ± standard deviation (*n* = 3), and different superscripted letters indicate significance at *P* < 0.05.

The improvement effect of BLW and IQ treatment on adipogenetic agent protein expression levels of FFA treated HepG2 cells was investigated using western blot evaluation. The protein expressions of CD36 and FAS were significantly increased in the only FFA treated group, the FFA treated with BLW or IQ groups showed significantly decreased levels compared to the only FFA treated group. The protein expressions of Sirt1, p-AMPK/AMPK, SREBP1c, p-ACL/ACL, p-ACC/ACC, PPAR-, and CPT1A were significantly decreased in the only FFA treated group compared to the NC group, while FFA treated with BLW or IQ groups showed a significant increase compared to the only FFA treated group (Fig. 8).

### Effects of BLW and IQ on protein expression of inflammation and apoptosis related agent in FFA treated HepG2 cells

**Fig. 9:**
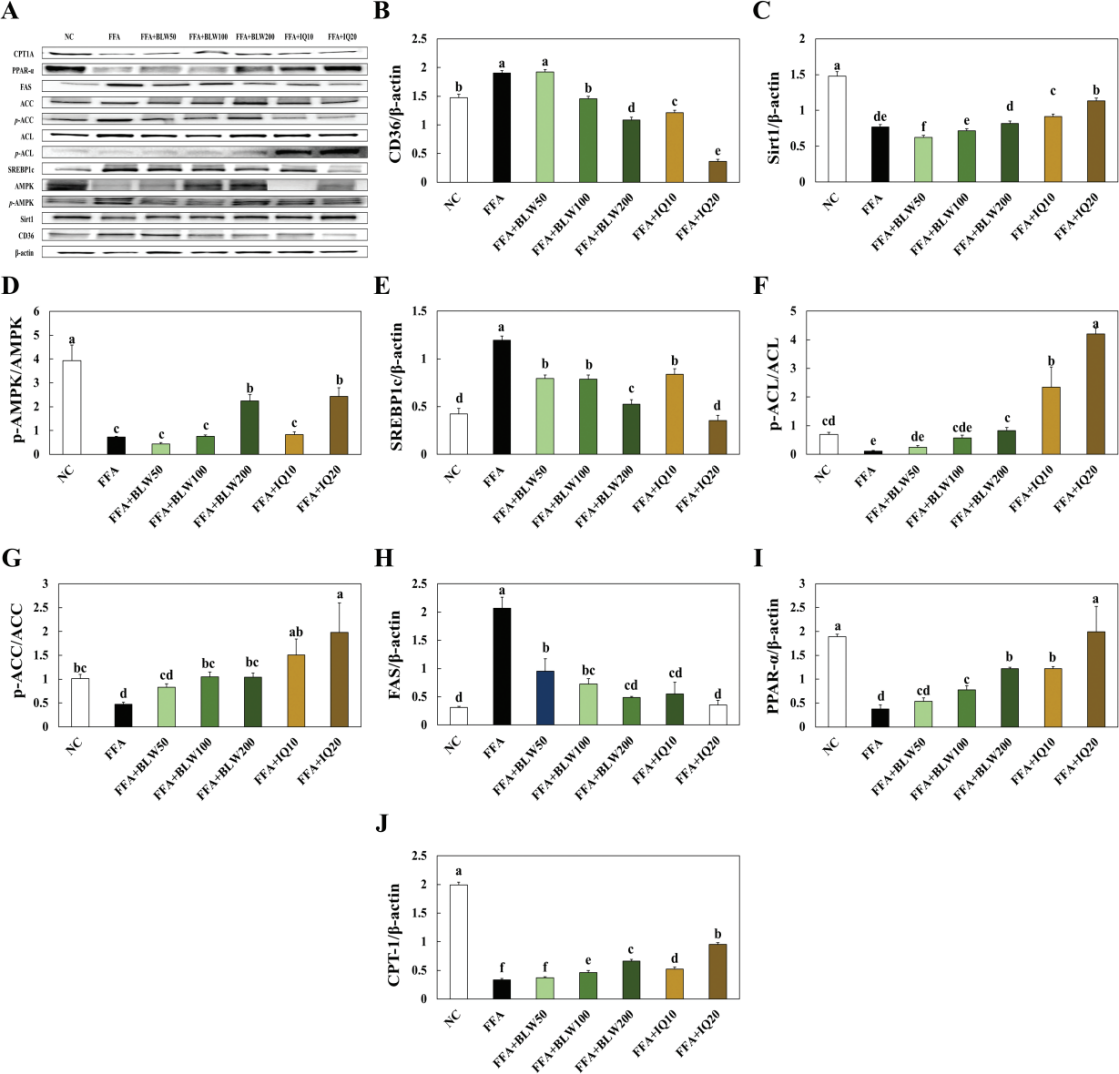
Effect of BLW and IQ on inflammation and apoptosis related protein expression in FFA treated HepG2 cell. (A) band, (B) p-IκB/IκB, (C) p-p65/p65, (D) COX-2, (E) iNOS, (F) p-JNK/JNK, (G) Bax/Bcl-2, (H) CD95, (I) FADD, (J) C-CAS8, (K) C-CAS3. NC, Normal control; C, 1 mM free fatty acid (FFA, 2:1 ratio for oleate:palmitate); FFA+BLW50, 50 μg/mL of BLW+1 mM FFA; FFA+BLW100, 100 μg/mL of BLW+1 mM FFA; FFA+BLW200, 200 μg/mL of BLW+1 mM FFA; FFA+IQ10, 10 μM of isoquercetin+1 mM FFA; FFA+IQ20, 20 μM of isoquercetin+1 mM FFA. Values are presented as the mean ± standard deviation (*n* = 3), and different superscripted letters indicate significance at *P* < 0.05.

To investigate the improvement effect of BLW and IQ treatment on protein expression related to the inflammation and apoptosis pathways, we analyzed p-IκB/IκB, p-p65/p65, COX-2, iNOS, p-JNK/JNK, BAX/Bcl-2, CD95, FADD, C-CAS8, and C-CAS3 protein expression levels of FFA treated HepG2 cells using western blot evaluation. The p-IκB/IκB ratio, p-p65/p65 ratio, COX-2, and iNOS expressions were significantly higher in the only FFA treated group than the NC group, while the FFA treated with BLW or IQ groups showed a decrease in those expressions. The apoptosis related proteins such as p-JNK/JNK ratio, BAX/Bcl-2 ratio, CD95, FADD, C-CAS8, and C-CAS3 were significantly increased in the only FFA treated group compared to the NC group. However, the FFA treated with BLW or IQ groups showed a decrease in those protein expressions compared to the only FFA treated group (Fig. 9).

## Discussion

The prevalence of NAFLD has increased dramatically along with an increase in prevalence of metabolic syndrome and obesity, which has been attributed to increased fat and fructose consumption in the global population. No pharmaceutical treatments for NAFLD have been approved, and existing studies have focused on the protective effects of natural foods on NAFLD (17, 18). A high fat diet induces lipid accumulation in adipocytes and hepatocytes and high levels of serum TG and LDL-cholesterol can result in accelerated development of atherosclerosis (19). We examined the effects of BLW in HFHF induced NAFLD in mice by employing ELISA and western blot analyses of serum and liver samples. BLW supplementation resulted in a significant reduction in hepatic lipid droplets compared with the HFHF group. Increased serum ALT, AST, and GGT levels indicate liver dysfunction; we found that BLW supplementation significantly decreased expression of these markers relative to the HFHF group, consistent with the results of previous studies (20, 21). Therefore, BLW showed decreased serum AST, ALT, GGT, and lipid profiles levels and the moderation of histopathological changes.

NAFLD is associated with ROS generated in the mitochondria of liver cells. Antioxidant enzymes such as SOD, CAT, and GPx catalyze the conversion of O_2_˙- to H_2_O_2_, and subsequent catalysis of H_2_O_2_ to O_2_ and H_2_O, GSH and H_2_O_2_ converting to H_2_O and GSSG (22–24). Hepatic SOD, CAT, and GPx activities were decreased in the HFHF group, while the activities of these enzymes were significantly increased in the BLW supplementation groups compared to the HFHF group. Fructose is a dietary carbohydrate which is metabolized in the liver over 90% unlike glucose. Fructose is absorbed from the intestinal lumen and the ingested fructose is transported into the liver via GLUT2/GLUT8. When energy is enough, glyceraldehyde-3 phosphate, which is produced via fructose-1-phosphate by several steps could be used in fatty acid synthesis (25–27). CD36 is the main plasma membrane transporter for FFA (9, 28). Our results demonstrated that BLW supplementation significantly reduced the protein expression of hepatic GLUT2 and CD36 compared to the HFHF group suggesting that BLW could inhibit the movement of fructose and FFA into hepatocytes. Intracellular fructose and FFA decrease Sirt1, AMPK, ACL, ACC, PPAR-α, and CPT1A activation and increase SREBP-1c and FASN expression, resulting in TG accumulation (29, 30). Phosphorylation of AMPK controls SREBP-1c expression, phosphorylation of ACL, ACC activity, as well as FASN expression. Furthermore, PPAR-α regulates the expression of CPT1A, which plays an essential role in fatty acid β-oxidation (31, 32). We found that BLW supplementation increased Sirt1, AMPK, ACL, ACC, PPAR-α, and CPT1A activation and decreased SREBP-1c and FASN expression, indicating that BLW can attenuate hepatic lipid accumulation. Excess ROS activate NF-κB pathways as an inflammatory response and the JNK pathway for apoptosis in hepatocytes (33, 34). In NAFLD patients, phosphorylation of IκB and NF-κB results in their translocation to the nucleus in hepatocytes, resulting in the expression of inflammatory genes, while phosphorylation of JNK (p-JNK) induces an increase in the BAX/Bcl-2 ratio, CD95, FADD, C-CAS8, and C-CAS3 expression (11, 12). BLW supplementation decreased the expression of proteins related to inflammation (p-IκB/IκB ratio, p-NF-κB/NF-κB ratio, COX-2, and iNOS) and apoptosis (p-JNK/JNK ratio, BAX/Bcl-2 ratio, CD95, FADD, C-CAS8, and C-CAS3) compared to the HFHF group.

Additionally, palmitic acid and oleic acid as well as a saturated fatty acid are one of the FFA circulating in the blood (35–37). Palmitic acid and oleic acid mixture led to lipid accumulation in HepG2 cells. Our study proved that the FFA mixture exhibited increased lipid accumulation by oil-red O staining while BLW and IQ pre-treatment showed a decreased lipid accumulation (38). Previous study has reported that IQ was decreased in lipid accumulation of rat hepatoma (H4IIE cells) induced by FFA (39). Lipid accumulation in the liver lead to oxidative stress. A previous paper reported that the level of SOD and the expression of HO-1 and Nrf2 was increased in FFA treated HepG2 cells (40). Hepatocyte apoptosis is an essential mechanism in the progression of NAFLD. Previous studies reported that FFA can lead to the activation of the key regulator of inflammatory cytokines causing JNK-dependent apoptosis. There are two pathways in apoptosis such as receptor mediated apoptotic pathways and cytotoxic stress (41, 42). Our research conducted cellular experiments to investigate the protective effects of BLW against NAFLD. The results demonstrated that the only FFA treated group exhibited typical NAFLD symptoms, including excessive lipid accumulation, inflammatory responses, and apoptosis. Conversely, the cells treated with a combination of FFA and either BLW or IQ showed improved symptoms. Notably, the cells treated with a combination of FFA and IQ, a known active component of BLW, demonstrated even more pronounced amelioration of NAFLD symptoms.

## Conclusions

Overall, our findings indicate that BLW and IQ attenuate hepatic lipid accumulation and inhibit hepatic inflammatory response and apoptosis pathways in hepatocytes in the context of NAFLD. However, further research is needed to elucidate how BLW and IQ increase the expression of antioxidant enzymes, through which specific pathways they act, and how they improve the expression of lipogenesis, inflammatory, and apoptosis-related molecules such as microRNAs and circular RNAs.
